# Supplementary material for: Sorting at embryonic boundaries requires high heterotypic interfacial tension
Source: Nat Commun. 2017 Jul 31;8:157. doi: 10.1038/s41467-017-00146-x (PMC5537356; doi:10.1038/s41467-017-00146-x)
Supplement: Supplementary file 2 — Supplementary Software 1 [file 41467_2017_146_MOESM2_ESM.zip › PottsModel/SrcPottsModel/doc/simulations/DefaultSimulation.html]

DefaultSimulation


JavaScript is disabled on your browser.


Skip navigation links


- Overview
- Package
- Class
- Use
- Tree
- Deprecated
- Index
- Help

- Prev Class
- Next Class

- Frames
- No Frames

- All Classes

- Summary:
- Nested |
- Field |
- Constr |
- Method

- Detail:
- Field |
- Constr |
- Method


simulations

## Class DefaultSimulation

- java.lang.Object
- - engine.Simulation
  - - simulations.DefaultSimulation

- ---

    

  ```
  public class DefaultSimulation
  extends Simulation
  ```

- - ### Constructor Summary

    Constructors

    Constructor and Description

    `DefaultSimulation()`
  - ### Method Summary

    All Methods Static Methods Instance Methods Concrete Methods

    | Modifier and Type | Method and Description |
    | --- | --- |
    | `Constants[]` | `getConstants()` |
    | `static void` | `main(java.lang.String[] args)` |

    - ### Methods inherited from class engine.Simulation

      `run`
    - ### Methods inherited from class java.lang.Object

      `equals, getClass, hashCode, notify, notifyAll, toString, wait, wait, wait`

- - ### Constructor Detail


    - #### DefaultSimulation

      ```
      public DefaultSimulation()
      ```
  - ### Method Detail


    - #### getConstants

      ```
      public Constants[] getConstants()
      ```

      Specified by:
      :   `getConstants` in class `Simulation`


    - #### main

      ```
      public static void main(java.lang.String[] args)
      ```


Skip navigation links


- Overview
- Package
- Class
- Use
- Tree
- Deprecated
- Index
- Help

- Prev Class
- Next Class

- Frames
- No Frames

- All Classes

- Summary:
- Nested |
- Field |
- Constr |
- Method

- Detail:
- Field |
- Constr |
- Method
